# The Effect of Intraoral Thermal Changes on the Mechanical Behavior of Nickel-Titanium Wires: An In-Vitro Study

**DOI:** 10.7759/cureus.72207

**Published:** 2024-10-23

**Authors:** Odayy S Al-Horini, Mariam Marwan Masaes, Feras Baba, Mohammad Y Hajeer, Soghyia Talib Ibrahim Almulla, Mudar Mohammad Mousa

**Affiliations:** 1 Department of Orthodontics, Faculty of Dentistry, University of Aleppo, Aleppo, SYR; 2 Department of Orthodontics, Faculty of Dentistry, University of Damascus, Damascus, SYR; 3 Department of Orthodontics, Sharjah Specialized Dental Center, Emirates Health Services, Sharjah, ARE

**Keywords:** austinite finish temperature, heat activated, niti archwires, residual strain, shape memory, thermal sensitivity, unloading forces

## Abstract

Background

The continuous light force with a wide range of activation describes the excellent properties of nickel-titanium (NiTi) archwires. Shape memory is mainly affected by intraoral thermal changes. This study evaluated the effect of three different constant temperatures (i.e., 12°C, 37°C, and 50°C) on the unloading value of three different 0.016 × 0.022 NiTi archwires.

Methodology

Three types of 0.016 × 0.022-inch diameter NiTi archwires (American Orthodontics®, Sheboygan, Wisconsin, USA) were used. These were the superelastic type (NT3-SE®), the heat-activated type at 25°C (Thermal Ti-D®), and the thermally activated type at 35°C (Thermal Ti-Lite®). The unloading forces of the wires were evaluated using a classic three-point bending test (a universal testing machine: Testometric 350M®, Instron, Lincoln Close, Rochdale, England) under three different constant temperatures (12°C, 37°C, and 50°C).

Results

All types of wires showed thermal sensitivity; at higher temperatures, the unloading forces increased differentially between small and large deflections, while at lower temperatures, the residual strain increased for all wire types. The most affected type by the thermal changes was thermal Ti-Lite®, followed by thermal Ti-D®, and the superelastic type NT3-SE® showed a behavior similar to thermal wires. At the low temperature (12°C), all wire types showed an incomplete load/deflection curve, whereas no value was measured at unloading points 2, 1, and 0.5 mm. At the normal temperature (37°C), NT3-SE® type and thermal Ti-D® were similar in force level, while significant differences were noted between both previous types and Thermal Ti-Lite®. At the high temperature (50°C), all wire types showed a higher force level, while significant differences between the wire types were inconsistent. In contrast, increasing the temperature from 37°C to 50°C increased the force levels between 40% and 84% for NT3-SE®, between 44% and 64% for the thermal Ti-D®, and between 61% and 268% for the Thermal Ti-Lite®. When comparing the force levels between 12°C and 50°C at 3 mm, the force levels increased by 66% for NT3-SE®, 25% for Thermal Ti-D®, and 109% for thermal Ti-Lite®, while on comparing the forces between 12°C to 37°C, the forces increased between 15% and 95% for NT3-SE®, 20% and 88% for thermal Ti-D®, and 26% and 78% for thermal Ti-Lite®. The value of residual strain was greater at low temperatures and smaller at higher temperatures, while no significant differences were detected between 37°C and 50°C.

Conclusions

The temperature degree deeply affected the mechanical behavior of all test NiTi wires; the superelastic type behaved similarly to thermal wires. Increasing the temperature degree leads to more unloading forces and less residual strain.

## Introduction

In modern orthodontics, nickel-titanium (NiTi) wires are considered the best choice during the leveling and alignment stages [1,2] due to their special ability to produce gentle, continuing forces over an extensive loading range [3-5]. This ability could represent the status of the crystalline structure and the temperature effect [6].

Since 1970, many generations of NiTi wires have appeared on the market, which require several classifications to determine the differences between the cables and the factors that affect their properties. The most commonly used classification is the one proposed by Kusy [2], who divided NiTi wires into the following three major groups: the first group included the conventional NiTi or non-elastic Nitinol archwires with minimum thermal sensitivity; the second group included the superelastic or pseudo-elastic NiTi archwires with moderate thermal sensitivity; and the third group included the thermoelastic or heat-activated NiTi archwires with high thermal sensitivity [2,4,5].

In general, the intraoral temperature varies depending on the physical situation, function, and type of diet [7]. During the day, the intraoral temperature changes between the maximum value in the afternoon and the minimum in the evening. It also varies according to the function of the masticatory system, from the chewing phase (active) to the resting phase (relaxation) [8]. Finally, the type of diet affects these changes significantly; a diet with hot food and drinks would raise the oral temperature, while a vegan diet decreases it [9].

These dramatic changes in oral temperature have a significant impact on the mechanical behavior of the NiTi archwires in two ways. The first is that if they raise the environmental temperature above the transformation temperature range (TTR) of the NiTi wire, it will produce a full austenitic transition, which, in turn, will increase the fatigue resistance of the NiTi wires. The second is that if they lower the temperature below the TTR of the NiTi wire, it will produce a martensitic transition, decreasing the fatigue resistance [5,6,10-12].

In light of some in-vitro studies, Lombardo et al. [5] studied the effect of temperature on the load/deflection curve of conventional and heat-activated NiTi wires from different brands and different cross-section diameters at 5°C and 55°C. They found that all NiTi wires showed a change in behavior and force for both traditional and heat-activated wires related to temperature changes, and a permanent strain occurred at high temperatures, whereas the residual strain at low temperatures was higher in value than that detected at high temperatures. While Iijima et al. [13] framed both the mechanical and microstructure of 0.016 × 0.022 conventional and heat-activated NiTi wires at constant (23°C-37°C and 60°C) and stepwise temperatures (37-60°C and to 37°C). They claimed that the force values at constant temperature were consistent with the value of the stepwise one and that the X-ray diffraction spectrum showed a crystalline transition when the temperature changed through the transformation range for each type of wire.

The current study aimed to evaluate the effect of three different constant temperatures (12°C, 37°C, and 50°C) on the unloading values of three different NiTi wires of rectangular 0.016 × 0.022-inch cross-section: the superelastic wire and the thermally heat-activated wires at 25°C and 35°C, respectively. The null hypothesis is that the tested NiTi archwires do not show any differences in released forces at the three different constant temperatures.

## Materials and methods

Study design and setting

This study was a single-blind, randomized, controlled in vitro study to investigate how three different temperature degrees affect the unloading value of three different NiTi wires. It was conducted at the University of Aleppo, Syria, in the Department of Orthodontics, Faculty of Dentistry, and Faculty of Mechanical Engineering.

Sample size calculation

In the study by Sakima et al. [14], which evaluated how temperature influences the properties of rectangular NiTi wires at a sample size of seven wires, the standard deviation was 6, and the main difference in the load forces between different temperatures was +1.38 N at a 95% confidence level. The sample size was calculated using Minitab® Version 17 (Minitab Inc., State College, Pennsylvania, USA), and six wires were found to be required for each group.

Wires used in the experiment

Three types of NiTi archwires were included. The first one was superplastic NiTi wires with moderate force levels (NT3-SE®, American Orthodontics, Sheboygan, Wisconsin, USA). The second was thermal NiTi wires activated over 25°C with moderate force levels (Thermal Ti-D®, American Orthodontics, Sheboygan, Wisconsin, USA). The third was thermally activated wires at 35°C (Thermal Ti-Lite®). All archwires were 0.016 × 0.022 inches in dimensions. The test was at a length of 30 mm, cut by a heavy straight cutter from the posterior section of the arch.

Experimental groups

**Figure 1:**
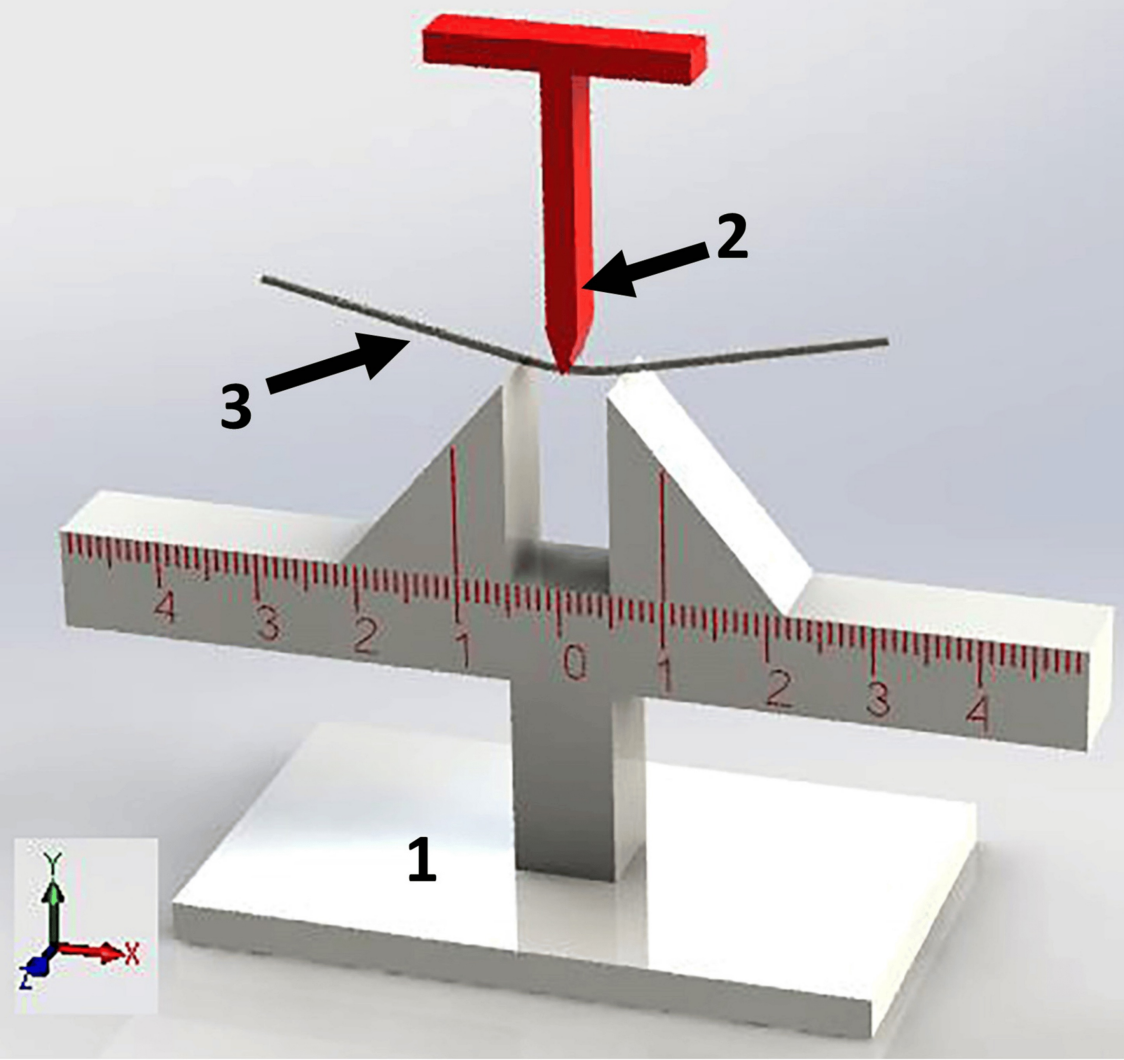
Simulation of the wire and the wire holder. 1: Plexiglas wire holder. 2: The crosshead used in the experiment. 3: The nickel-titanium (NiTi) archwire.

The first group (low temperature) included the three types of wires tested at 12°C, whereas the second group (norm temperature) included the three types tested at 37°C. The third group (high temperature) included the three types of wire tested at 50°C by the bending test (Figure 1).

Outcome measurement: load/deflection curves

**Figure 2:**
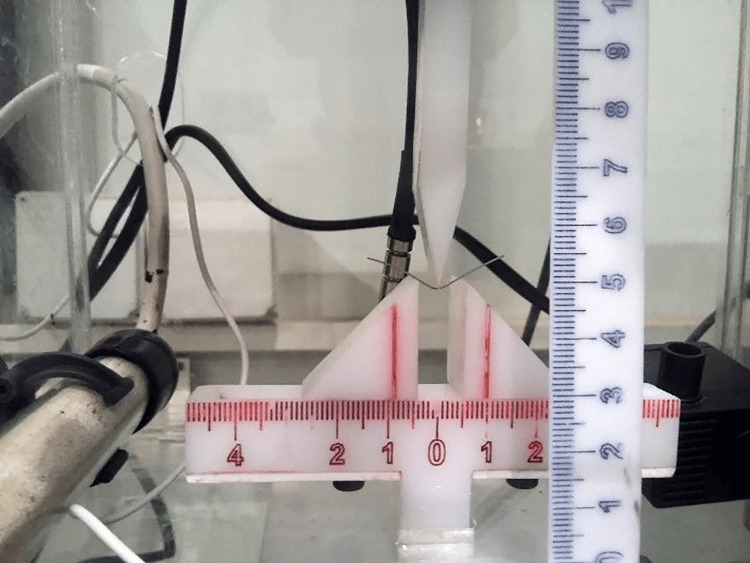
The water bath used to conduct the three-point bending test with a constant temperature of about 37°C.

The classic three-point bending test described utilizes a universal testing machine (Testometric 350M®, Instron, Lincoln Close, Rochdale, England) equipped with a modified 100 N load cell and a Plexiglas water bath with a water pumper to ensure mixing the water inside the bath, a thermostat to monitor the temperature changes, and a water heater. For 37°C and 50°C, the water heater was used to increase water temperature from room temperature 25°C to the required degree. For 12°C, cold water was added (5°C) to the water bath and warmed till 12°C (Figure 2) [10,15]. The setup involved testing wires of 30 mm in length with a span of 10 mm, where the crosshead deflected the wire by 3.1 mm at a rate of 1 mm/minute, ensuring point contact at the wire’s center. The vertical deflection was measured in the occlusal-gingival direction, adhering to the ISO/DIS 15841 guidelines to evaluate the mechanical properties of orthodontic wires [16].

The resulting load/deflection curves were analyzed using Solid Works® version 2012 software (SolidWorks®, Dassault systems, Concord, Massachusetts, USA) and Microsoft Excel (Microsoft Excel™ 2013, Microsoft Corp., Redmond, Washington, USA). The unloading forces were evaluated at the following five points: 0.5, 1, 2, 3 mm, and residual strain. These points expressed the deflection of wire and the amount of force that the tooth would receive during the start (i.e., 3 mm) and the end (i.e., 0.5 mm) of the unloading stage; the residual strain expressed the difference between the liner loading and liner unloading stage.

Statistical analysis

Statistical analysis was conducted using IBM SPSS Statistics for Windows Version 20.0 (IBM Corp., Armonk, NY, USA). Kolmogorov-Smirnov tests were employed to evaluate the normality of data distributions. For the analysis of variance, a one-way analysis of variance test was utilized, followed by post-hoc Sidak’s test to identify significant differences between each pair of groups, with a significance level set at 5%

Error of the used method

The method’s reliability was checked by evaluating the correlation coefficient of the three wires chosen randomly from the tested sample. Pearson’s correlation coefficient performed well, and the result was a high value of the Pearson bivariate test (0.866), which indicated a good level of the method’s reliability.

## Results

Effect of temperature degrees

Effect of Low Temperature (12°C)

**Figure 3:**
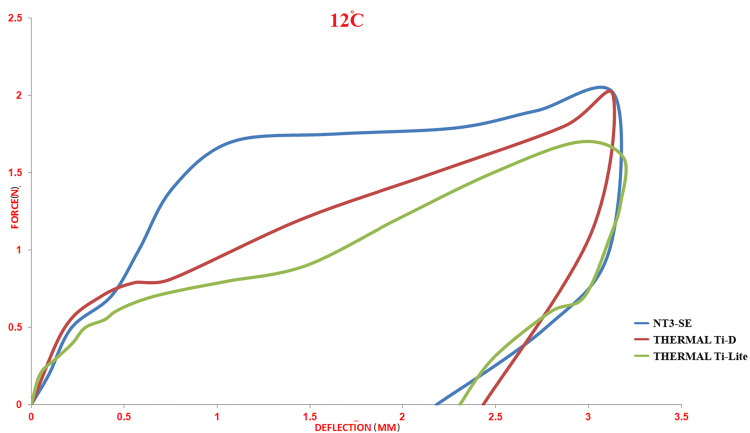
Load/deflection curves of the three different nickel-titanium archwires at a stable temperature of 12°C.

All three wire types showed an incomplete load/ deflection curve, where the loading curve appeared, but the unloading curve did not appear (Table 1; Figure 3). Regarding the statistical differences, only one significant difference was found between the superelastic type (NT3-SE®) and the thermally activated at 25°C (Thermal Ti-D®), with the thermally activated at 35°C (Thermal Ti-Lite) at the unloading point 3 mm (p < 0.001) while no value measured at 2, 1, 0.5 mm (Table 2).

**Table 1:** Descriptive statistics of the unloading forces for the three wire types regarding the three different temperatures. SD: standard deviation; Max: maximum; Min: minimum; NT3-SE: superelastic type; Thermal Ti-D: heat-activated type at 25°C; Thermal Ti-Lite: heat-activated type at 35°C

Temperature	Unload value	Wire type											
		NT3-SE^®^				Thermal Ti-D^®^				Thermal Ti-Lite^®^			
		Mean	SD	Max	Mini	Mean	SD	Max	Mini	Mean	SD	Max	Mini
12°C	0.5 mm	0.0	0.0	0.0	0.0	0.0	0.0	0.0	0.0	0.0	0.0	0.0	0.0
	1 mm	0.0	0.0	0.0	0.0	0.0	0.0	0.0	0.0	0.0	0.0	0.0	0.0
	2 mm	0.08	0.10	0.25	0.0	0.16	0.10	0.28	0.0	0.16	0.10	0.29	00
	3 mm	2.24	0.14	2.5	2.11	2.07	0.11	2.25	1.95	1.62	0.10	1.80	1.50
	Residual strain	2.08	0.14	2.3	1.90	2.15	0.18	2.4	1.9	2.16	0.10	2.3	2.0
37°C	0.5 mm	1.09	0.19	1.35	0.85	0.89	0.15	1.10	0.7	0.38	0.20	0.70	0.15
	1 mm	1.40	0.18	1.15	1.65	1.25	0.9	1.45	1.10	0.52	0.09	0.65	0.40
	2 mm	1.60	0.18	1.4	1.95	1.44	0.11	1.60	1.30	0.76	0.10	0.90	0.60
	3 mm	2.65	0.11	2.5	2.8	2.50	0.11	2.65	2.35	2.10	0.15	2.35	1.90
	Residual strain	0.07	0.06	0.14	0.0	0.15	0.03	0.19	0.11	0.27	0.04	0.35	0.23
50°C	0.5 mm	2.01	0.08	2.10	1.90	1.70	0.10	1.80	1.55	1.41	0.14	1.60	1.20
	1 mm	2.10	0.07	2.20	2.05	1.94	0.11	2.10	1.80	1.76	0.15	1.90	1.50
	2 mm	2.26	0.14	2.90	2.50	2.34	0.14	2.60	2.20	2.38	0.19	2.70	2.20
	3 mm	3.71	0.27	4.00	3.20	3.59	0.17	3.80	3.30	3.39	0.13	3.60	3.25
	Residual strain	0.65	0.07	0.14	0.00	0.56	0.06	0.12	0.00	0.05	0.06	0.13	0.00

**Table 2:** Post-hoc tests for comparisons between the three archwire types at the three temperature degrees (12°C, 37°C, and 50°C). *: significant at p < 0.05. NT3-SE: superelastic type; Thermal Ti-D: heat-activated type at 25°C; Thermal Ti-Lite: heat-activated type at 35°C

Temperature	Unload value	Wire type	P-value
12°C	2 mm	NT3-SE® * Thermal Ti-D®	0.472
		NT3-SE® * Thermal Ti-light®	0.489
		Thermal Ti-D® * Thermal Ti-light	1.000
	3 mm	NT3-SE® * Thermal Ti-D®	0.082
		NT3-SE® * Thermal Ti-light®	<0.001*
		Thermal Ti-D® * Thermal Ti-light®	<0.001*
	Residual strain	NT3-SE® * Thermal Ti-D®	0.839
		NT3-SE® * Thermal Ti-light®	0.730
		Thermal Ti-D® * Thermal Ti-light®	0.997
37°C	0.5 mm	NT3-SE® * Thermal Ti-D®	0.241
		NT3-SE® * Thermal Ti-light®	<0.001*
		Thermal Ti-D® * Thermal Ti-light®	<0.001*
	1 mm	NT3-SE® * Thermal Ti-D®	0.170
		NT3-SE® * Thermal Ti-light®	<0.001*
		Thermal Ti-D® * Thermal Ti-light®	<0.001*
	2 mm	NT3-SE® * Thermal Ti-D®	0.222
		NT3-SE® * Thermal Ti-light®	<0.001*
		Thermal Ti-D® * Thermal Ti-light®	<0.001*
	3 mm	NT3-SE® * Thermal Ti-D®	0.197
		NT3-SE® * Thermal Ti-light®	<0.001*
		Thermal Ti-D® * Thermal Ti-light®	0.003*
	Residual strain	NT3-SE® * Thermal Ti-D®	0.057
		NT3-SE® * Thermal Ti-light®	<0.001*
		Thermal Ti-D® * Thermal Ti-light®	<0.001*
50°C	0.5 mm	NT3-SE® * Thermal Ti-D®	<0.001*
		NT3-SE® * Thermal Ti-light®	<0.001*
		Thermal Ti-D® * Thermal Ti-light®	0.002*
	1 mm	NT3-SE® * Thermal Ti-D®	<0.001*
		NT3-SE® * Thermal Ti-light®	0.074
		Thermal Ti-D® * Thermal Ti-light®	0.058
	2 mm	NT3-SE® * Thermal Ti-D®	<0.001*
		NT3-SE® * Thermal Ti-light®	0.026*
		Thermal Ti-D® * Thermal Ti-light®	0.962
	3 mm	NT3-SE® * Thermal Ti-D®	0.661
		NT3-SE® * Thermal Ti-light®	0.042*
		Thermal Ti-D® * Thermal Ti-light®	0.291
	Residual strain	NT3-SE® * Thermal Ti-D®	0.995
		NT3-SE® * Thermal Ti-light®	0.998
		Thermal Ti-D® * Thermal Ti-light®	1.000

Effect of Normal Temperature (37°C)

**Figure 4:**
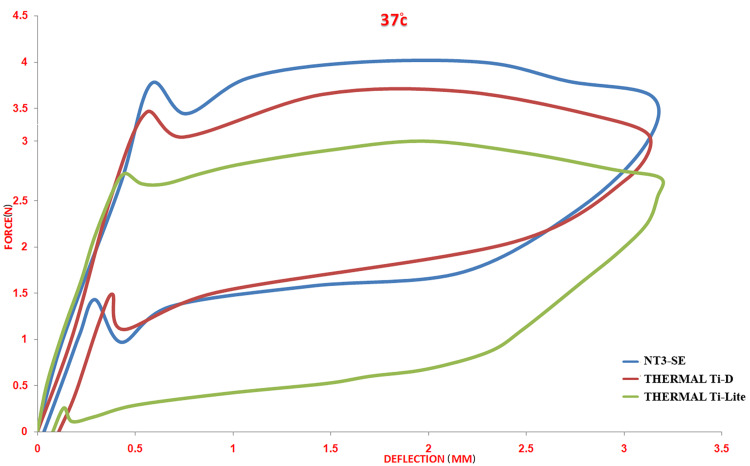
Load/deflection curves of the three different nickel-titanium archwires at a stable temperature of 37°C.

A classic load/deflection curve for the three different wire types was observed when the test was done at normal temperature (37°C). The superelastic type (NT3-SE®) was almost similar in all measurement points to the thermal type activated at 25°C (Thermal Ti-D®), while both types had a significant difference from the thermal type activated at 37°C (Thermal Ti-Lite®) for all unloading points (p = 0.005) (Table 2; Figure 4).

Effect of High Temperature (50°C)

**Figure 5:**
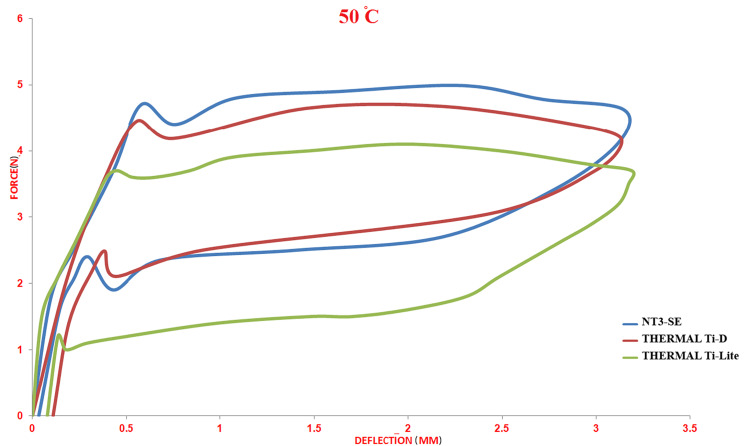
Load/deflection curves of the three different nickel-titanium archwires at a stable temperature of 50°C.

The load/deflection curve was almost similar to the classic curve, with the highest force levels for all wires. However, the pattern of significant differences between the three wires was inconsistent. At the unloading point of 3 mm, there was a significant difference between the NT3-SE® and the Thermal Ti-Lite® wires (p = 0.042), whereas, at the unloading point of 2 mm, a difference was detected between the NT3-SE® and both thermally activated types (Thermal Ti-D® and Thermal Ti-Lite®; p < 0.001 and p = 0.026, respectively). At the 1 mm unloading point, a significant difference was only detected between the NT3-SE® and the Thermal Ti-D® wires (p < 0.001). Finally, at the unloading point of 0.5 mm, significant differences were found between all wire comparisons (Table 2; Figure 5).

Effect of wire type

Raising the temperature from 37°C to 50°C increased the force levels between 40% and 84% for the superelastic type (NT3-SE®), 44% and 64% for the thermally activated type at 25°C (Thermal Ti-D®), and between 61% and 268% for the thermally activated type at 35°C (Thermal Ti-Lite®), while the residual strain decreased respectively (Table 1). Decreasing the temperature from 37°C to 12°C reduced the force levels for unloading points at 3 and 2 mm by 15% and 95% for the superelastic type (NT3-SE®), 20% and 88% for the thermally activated type at 25°C (Thermal Ti-D®), and between 26% and 78% for the thermally activated at 35°C (Thermal Ti-Lite®), respectively, while the residual strain increased greatly at lower temperature for all types (Table 1).

Comparing the values between 12°C and 50°C, the mean values were greater for the higher temperature at the unloading point (3 mm) by 66% for the superelastic type (NT3-SE®), 25% for the thermally activated type at 25°C (Thermal Ti-D®), and by 109% for the thermally activated type at 35°C (Thermal Ti-Lite®), while the residual strain increased greatly at lower temperature for all types (Table 1).

**Figure 6:**
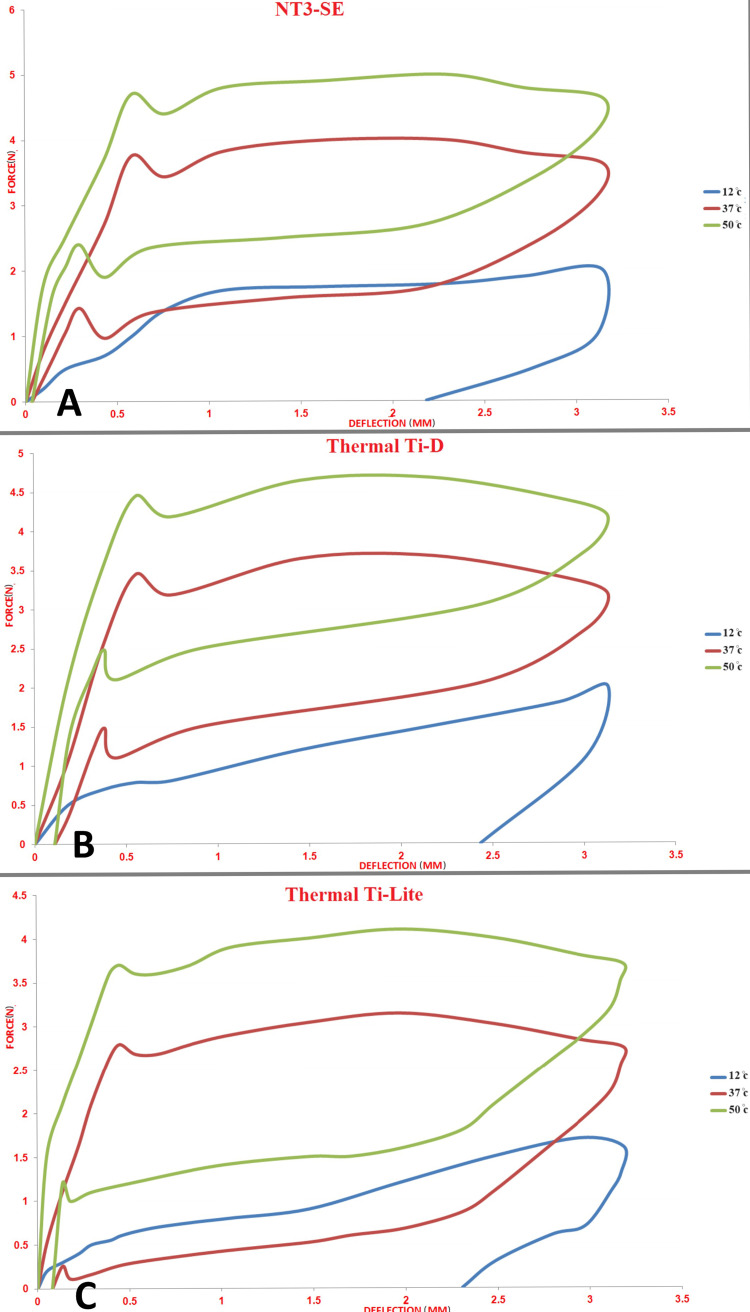
Load/deflection curves at the three different temperatures of 12°C, 37°C, and 50°C for the tested archwires. A: NT3-SE®, B: Thermal Ti-D®, C: Thermal Ti-Lite®.

Significant differences were observed at all tested points for all wire types at the three different temperature degrees except for the thermally activated (Thermal Ti-D®) and superelastic (NT3-SE®) types, in which there was no significant difference in residual strain between 37°C and 50°C (Table 3; Figure 6).

**Table 3:** Post-hoc tests for comparisons between the three temperatures for the three archwires. NT3-SE: superelastic type; Thermal Ti-D: heat-activated type at 25°C; Thermal Ti-Lite: heat-activated type at 35°C

Wire types	Unload value	Comparing temperatures	P-value
NT3-SE®	0.5mm	37°C * 50°C	<0.001
		37°C * 12°C	0.007
		50°C * 12°C	<0.001
	1 mm	37°C * 50°C	<0.001
		37°C * 12°C	<0.001
		50°C * 12°C	<0.001
	2 mm	37°C * 50°C	<0.001
		37°C * 12°C	<0.001
		50°C * 12°C	<0.001
	3 mm	37°C * 50°C	<0.001
		37°C * 12°C	0.007
		50°C * 12°C	<0.001
	Residual strain	37°C * 50°C	NC
		37°C * 12°C	<0.001
		50°C * 12°C	<0.001
Thermal Ti-D®	0.5 mm	37°C * 50°C	<0.001
		37°C * 12°C	<0.001
		50°C * 12 °C	<0.001
	1 mm	37°C * 50°C	<0.001
		37°C * 12°C	<0.001
		50°C * 12°C	<0.001
	2 mm	37°C * 50°C	<0.001
		37°C * 12°C	<0.001
		50°C * 12°C	<0.001
	3 mm	37°C * 50°C	<0.001
		37°C * 12°C	<0.001
		50°C * 12°C	<0.001
	Residual strain	37°C * 50°C	NC
		37°C * 12°C	<0.001
		50°C * 12°C	<0.001
Thermal Ti-lite®	0.5 mm	37°C * 50°C	<0.001
		37°C * 12°C	<0.001
		50°C * 12°C	<0.001
	1 mm	37°C * 50°C	<0.001
		37°C * 12°C	<0.001
		50°C * 12°C	<0.001
	2 mm	37°C * 50°C	<0.001
		37°C * 12°C	<0.001
		50°C * 12°C	<0.001
	3 mm	37°C * 50°C	<0.001
		37°C * 12°C	<0.001
		50°C * 12°C	<0.001
	Residual strain	37°C * 50°C	<0.001
		37°C * 12°C	<0.001
		50°C * 12°C	<0.001

## Discussion

For most orthodontists, the NiTi wire is the best choice to start orthodontic treatment because it produces gentle, continuous forces among an extensive loading range [1,5,6]. This ability returns to the two important properties of NiTi wires: superelasticity and shape memory. Both refer to the changing crystalline structure between the austenite phase at high and low temperatures and the martensite phase at low temperatures and high stress [4,6].

The difference between the two terms is the role of temperature in producing those changes. In contrast, shape memory reflects the transition phase that mainly occurs under the effect of temperature changes in the TTR. In contrast, superelastics reflect the transition mainly under stress changes at constant temperature. In the market, NiTi wires are available in two major groups: the superelastic type and the thermally activated type. The first group is usually unaffected by thermal changes, while the second is mainly affected by temperature changes [6,10,17,18].

The bending test is one of the best mechanical methods to study the properties of NiTi wires [6,10,13]. The result of this test appears as a loading/unloading curve. The loading curve reflects the forces that were applied to the wire to be bent, while the unloading curve reflects the forces exerted by the wire itself. The NiTi wires have a specific shape that can be distinguished by the flattening in both turns [6,10,15].

In some special situations, the unloading curve disappears, which means the wire cannot return to its original shape after removing the applied forces. In other words, an irreversible deformity happens [14], which stops orthodontic movement due to the absence of forces applied from the wires to the tooth [6]. The disappearance of the unloading curve of NiTi wires could happen in two circumstances. The first is when a high level of stress is applied to the wires, which can break the atomic junction forces and even break the wire itself [11,12]. The second is when the temperature of the test is lower than the austenite finish temperature, which means the wires have a martensitic deformity, or, in other words, a failure of the reverse transition between the martensite and austenite phases [17,18]. In both situations, the disappearance of the unloading curve is called, in mechanical science, the residual strain [19]. Moreover, it can be detected on complete loading/unloading curves. In this situation, it reflects the invisible amount of deformity that NiTi wires exhibit [14].

Differential scanning calorimetry is one of the thermal analysis methods used to determine the TTR, which provides important and high-quality information in determining the austenite finish temperature [6,10,13,17,18]. As a general rule, binding a NiTi wire below that temperature will cause the reverse transition from martensite to austenite to decline, especially for the thermally activated types, while over that temperature, the reverse transition will increase [5,10,14]. In this study, a classic three-point bending test was performed for three different types of NiTi wires. Two of them were thermally activated at two different temperatures (thermal Ti-D and thermal Ti-Light), while the other type was superelastic (Nt3-SE). All wires had the same dimension (0.016 × 0.022) and were from the same manufacturer.

The bending tests were done at three different temperatures: 12°C, 37°C, and 50°C. The load/deflection curve was evaluated for each type at four points: 3, 2, 1, and 0.5 mm. The residual strain was calculated from the unloading curve. Regarding our previous investigation of NiTi wires’ mechanical and thermal behavior [10], the TTR for each type of wire denotes the austenite finish temperature of each type of wire.

Effect of low temperature (12°C)

All wire types showed an incomplete load/deflection curve when the test temperature was set at 12°C, whereas this temperature was lower than the austenite finish temperature for each type [10]; hence, the crystalline structure was completely in the martensite phase and reverse transition no longer occurred [6,10,13], which means no forces were applied from the wires to the teeth. At small deflections (0.5-1-2) mm, the value of residual strain was very convergent.

The only significant difference observed at a deflection of 3 mm between the thermally activated 35°C and the two other types could be explained by the differences in the value of the austenite finish temperature for each type. As an outline, at 12°C, the test temperature was lower than the austenite finish temperature for each type of wire, so a martensite deformity was recorded that started from the point of deflection of 2 mm, and a high amount of residual strain was noticed.

Effect of normal temperature (37°C)

A classic load/deflection curve was recorded for all wire types, which means the wires at this temperature exhibited a full turn of transition and reverse transition [6,10,19].

In comparing the unloading values between the wires, both types of wire, NT3-SE® and Thermal Ti-D®, had a convergent value for all studied unloading points. In contrast, the other thermal type, Thermal Ti-Light®, had significant differences with the previous types for all studied points that could be explained by the disparity in the austenite finish temperature between the wires. In contrast, the wires with a convergent austenite finish temperature value had approximated unloading values, concerning the value of NT3-SE® being slightly higher than that of Thermal Ti-D®.

Regarding the residual strain, the superelastic type showed the smallest value, followed by Thermal Ti-D®, which almost doubled, while Thermal Ti-Light® had the highest strain. As an outline, the superelastic types at normal temperature showed the latest unrecovered deformity, while both the thermal types showed a higher value instead. Further, significant differences were noticed for residual strain between NT3-SE and Thermal Ti-D® with Thermal Ti-light®.

Effect of high temperature (50°C)

All wire types exhibited a near-classic load/deflection curve, with increases at all unloading points that were the exception to the effect of raising the temperature over the austenite finish temperature [6,10,19].

When comparing the unloading value between the wires at 50°C as a set temperature test, the superelastic type exhibited the greatest released forces, followed by the Thermal Ti-D®, and, finally, the Thermal Ti-Light®. Significant differences were recorded between all the wires, and it randomly did not follow a determined distribution, e.g., for unloading point 3 mm, the superelastic type showed the highest value, followed by the thermal Ti-D, with significant differences noticed between the two previous types and the Thermal Ti-Light®. While for unloading point 2 mm, the significant differences were noticed between the superelastic type (NT3-SE®) and the two other thermal wires type (Ti-D® and Ti-Light®). For the residual strain, the value was approximately the same, which could lead us to conclude that raising the temperature test over austenite finish temperatures could reduce the unrecovered deformity and give more continuous forces even for small deflections. As an outline, at the test temperature over the austenite finish temperature, the unloading forces were increased at all unloading points for all wire types, but instead of that, the residual strain was almost equal; hence, raising the temperature over the austenite finish temperature led to a decrease in the residual strain.

Effect of archwire types

Superelastic Type (NT3-SE®)

At the beginning of the NiTi development journey, the first superelastic type, known as chaises NiTi or Japanese NiTi, showed some effect by chaining the temperature [1-6]. In comparing the unloading forces for the superelastic type at three different temperatures, many changes were noticed, and those changes were segmented into three minor groups.

The first one was between 37°C and 50°C. Raising the temperature raised the unloading forces between 40% and 80% for large deflection and reduced the residual strain by 17%. These changes led us to conclude that increasing the temperature for the superelastic type raised the unloading forces for both small and large deflection and reduced the total deformity of the wire. In other words, it made the wire more useful even at small deflection.

For the second group, between 37°C and 12°C, the forces were reduced by 15% for the large deflection and by 95% for the smaller deflection. The residual strain was increased by 2.5 mm, which led us to conclude that reducing the temperature would make the wire softer and stop the orthodontic movements.

For the third group between 12°C and 50°C. Raising the temperature raised the unloading forces by 66% for the large deflection and 96% for the smaller deflection, while the residual strain was still at the highest value for the lower temperature. To conclude the effect of temperature degree on the superelastic wires, the unloading forces were directly proportional to increasing the temperature while the residual strain dropped. In other words, this type of wire had a behavior similar to the thermal types, as reported by Lombardo et al. [5] and Sakima et al. [14]. It was likely related to a change in the manufacturing methods or the elemental composition. Finally, our conclusion for this type is compatible with many authors who found a tendency in the newest superelastic wires to behave like the thermal types [5,19].

Heat-Activated Type at 25°C (Thermal Ti-D®)

At the first marketing of heat-activated NiTi, the suppliers claimed that the wires could behave in two situations depending on the temperature: soft at low temperatures or hard at high temperatures [1,2,6]. Later, many studies focused on determining the specific temperature degree over which the wire could be stiffer, and below it, the wire could be softer [6,10,17-19]. That degree is the austenite finish temperature. Regarding our previous study [10], the austenite finish temperature was 23°C for this wire type.

This type of wire also showed differences in the mechanical behavior at the three different temperatures, whereas increasing the temperature between 37°C and 50°C made the wire stiffer, which increased the unloading forces by 47% for small deflection and by 44% for large deflection, while the residual strain was reduced by 62%. These changes were the best example of increasing the temperature over the austenite finish temperature on the mechanical behavior. While reducing the temperature between 37°C and 12°C dropped the unloading forces by 20% for large deflection and 88% for small deflection, only the residual strain increased by 2.00 mm, meaning the wire was soft and no orthodontic movement was expected.

Raising the temperature between 12°C and 50°C in the transition temperature range raised the unloading forces by 65% for large deflection, while the residual strain was significantly reduced. To conclude the effect of changing the temperature on the mechanical behavior of Thermal Ti-D, reducing the temperature over the austenite finish temperature made the wire stiffer, resulting in higher unloading forces and minimizing the residual strain, while lowering the temperature had an opposite effect that was accepted by many studies on the behavior of thermal wires [5,6,10,13,14,18].

Heat-Activated Type at 35°C (Thermal Ti-Light®)

The first generation of HANT wires was soft at low temperatures and stiffer at higher temperatures [1,2,6]. In material science, it was known before the development of NiTi alloy that some composite materials could react in different ways according to their crystalline structure [6]. The uniqueness of heat-activated wires was their ability to lower the transition temperature; hence, at small temperature changes, the wires could have a full transition and have two different reactions to the same action [6,19].

The differences in the transition temperature degree were attributed to the differences in elemental composition [20]. Regarding our previous study, the austenite finish temperature for Thermal Ti-Light® was 33°C [10]. A different reaction was recorded when the results of unloading forces were compared at the three different tested temperatures. Between 37°C and 50°C, the unloading forces were raised by 61% for large deflection and 240% for small deflection, while the residual strain decreased considerably by 81%.

Between 37°C and 12°C, the unloading forces decreased by 26% for large deflection and by 78% for small deflection, and the residual strain increased by 2 mm. Between 12°C and 50°C, the forces were raised by 95%, while the residual strain was higher at the lower temperature by 2.1 mm.

In conclusion, the Thermal Ti-Light was stiffer when the temperature was high and was softer at lower temperatures, while the residual strain was higher at low temperatures. Raising the temperature through the transition temperature would increase the unloading forces and reduce the residual strain [5,6,10,13,14,18].

Limitations of the current work

The limitations of the current study can be summarized in three points. The first is that the mechanical method used in the study was an Instron-350M device, the load cell was modified to be sensitive to lighter forces. This modification could lead to some outlier values. The second concerns the dependence on raising the temperature from a lower degree to a higher one because we did not have enough equipment to reduce the temperature from a higher degree to a lower one. The third was a single company that manufactured all the evaluated archwires. Therefore, future studies must focus on an unbiased and reliable assessment of various archwires from different manufacturers.

## Conclusions

The temperature considerably affected the mechanical behavior of all test NiTi wires. At 37°C, all wires showed a reverse transition, but the thermal types showed more unrecovered deformity than the superelastic types. While increasing the temperature to 50°C made all wires stiffer, which caused a higher unloading value, the residual strain was equal between all wires at this temperature. On the other hand, lowering the temperature to 12°C decreased the reversal transition and increased the residual deformity.

The unloading forces for the superelastic type (NT3-SE) increased when temperature increased by 80% to 96% for small deflection and by 15% to 66% for large deflection, while the residual strain was at the highest value at a low temperature of 12°C (2.5 mm) and the lowest value at a higher temperature of 50°C; hence, this wire behaves similar to thermal wires. Increasing the temperature leads to more unloading forces and less residual strain. At the same time, the thermal types (thermal Ti-D and thermal Ti-Lite) showed more thermal sensitivity for thermal change, especially at the small deflection. The forces increased by 240% for the thermal Ti-Lite and by 88% for thermal Ti-D, while at large deflection, the forces increased by 95% for the thermal Ti-Lite and by 20% for the thermal Ti-D, while the residual strain decreased. Finally, all the wire types show changes in force levels and residual strain. The small deflection was more affected by thermal changes than the large deflection.
